# Omni-Directional Scanning Localization Method of a Mobile Robot Based on Ultrasonic Sensors

**DOI:** 10.3390/s16122189

**Published:** 2016-12-20

**Authors:** Wei-Yi Mu, Guang-Peng Zhang, Yu-Mei Huang, Xin-Gang Yang, Hong-Yan Liu, Wen Yan

**Affiliations:** 1 Department of Mechanical and Precision Instrument Engineering, Xi’an University of Technology, Xi’an 710048, China; gpzhang@xaut.edu.cn (G.-P.Z.); hymxaut@163.com (Y.-M.H.); yxingang@163.com (X.-G.Y.); hyliu@xaut.edu.cn (H.-Y.L.); 2 Department of Art and Design, Xi’an University of Technology, Xi’an 710048, China; yanwen@xaut.edu.cn

**Keywords:** ultrasonic sensor, mobile robot, divergence angle and inclined angle, scanning localization, omni-directional localization

## Abstract

Improved ranging accuracy is obtained by the development of a novel ultrasonic sensor ranging algorithm, unlike the conventional ranging algorithm, which considers the divergence angle and the incidence angle of the ultrasonic sensor synchronously. An ultrasonic sensor scanning method is developed based on this algorithm for the recognition of an inclined plate and to obtain the localization of the ultrasonic sensor relative to the inclined plate reference frame. The ultrasonic sensor scanning method is then leveraged for the omni-directional localization of a mobile robot, where the ultrasonic sensors are installed on a mobile robot and follow the spin of the robot, the inclined plate is recognized and the position and posture of the robot are acquired with respect to the coordinate system of the inclined plate, realizing the localization of the robot. Finally, the localization method is implemented into an omni-directional scanning localization experiment with the independently researched and developed mobile robot. Localization accuracies of up to ±3.33 mm for the front, up to ±6.21 for the lateral and up to ±0.20° for the posture are obtained, verifying the correctness and effectiveness of the proposed localization method.

## 1. Introduction

The types of sensors used in the localization of mobile robots include laser sensors [1,2,3], visional sensors [4,5,6], infrared sensors [7], RFID (Radio Frequency Identification Devices) [8] and ultrasonic sensors [9,10], which compared with other sensors is the most robust and low-cost distance detection device [11]. The sound waves emitted by an ultrasonic sensor encompasses a fan-shaped area, the angle of which is defined as the divergence angle, and all objects that fall within this region can be detected. The distance accuracy of the ultrasonic sensor may be limited by failure to consider the divergence angle and the incidence angle, which refers to the angle between the cross-section of the ultrasonic sensor and the plane of the object being detected.

Song and Tang [12] reduced the impact of the divergence angle on the localization accuracy of a mobile robot by the application of external and independent Kalman filtering and the use of two ultrasonic sensors and a CCD (Charge-Coupled Device) vision sensor. Noykov and Roumenin [11] experimentally outlined an orientational probability graph for the divergence angle of an ultrasonic sensor and proposed an ultrasonic sensor edge detection method based on polaroid ultrasonic sensors. Kim and Kim [10] put forward a dual-ultrasonic sensor overlapping area distance detection method, which effectively decreased the influence of the divergence angle on the ranging accuracy of ultrasonic sensors, allowing for the precise localization of the posture of the car-like robot. Bin Liang et al. [13] brought forward the lateral localization method that employed two ultrasonic sensors installed on one side of the robot and considered the incident angle. Wijk and Christensen [14] proposed the use of information fusion technology for the indoor robot localization by the recognition of the fixed object. Carinena et al. [15] applied the novel paradigm of fuzzy temporal rules to detect doors using the information of ultrasonic sensors. Hwang et al. [16] introduced a simple GPS system for the indoor localization of a mobile robot that consisted of one transmitter having ultrasonic and RF (Radio Frequency) and two receivers. Li et al. [17] developed an ultrasonic sensor array heuristic controller system with group-sensor firing intervals, which was used to obtain the posture of a mobile robot in a parking space, and to ensure the ability to withstand collision and to guarantee safety parking. Kim and Kim [18] presented the optimal arrangement of an ultrasonic sensor ring with beam overlap for high resolution obstacle detection and minimal position uncertainty of a mobile robot. Hsu et al. [19] proposed a localization method based on the omni-directional ultrasonic sensor, which included a mobile robot carrying an omni-directional ultrasonic device as a transmitter and several ultrasonic sensors located at the vertices of a square environment serving as receivers. Lim et al. [20] proposed a novel control architecture which enabled a robot to navigate indoor environments while avoiding obstacles and localizing its current position by using a smartphone as its brain to deal with the heavy-duty and rotating ultrasonic sensors, reducing the number of sensors needed, as well as the time of distant measurements. Currently, research in the field of mobile robot localization is typically limited to uni-directional localization, for instance, forward or lateral localization, and research on the simultaneous forward, lateral and posture localization is rare.

Previous studies to reduce the effects of the divergence angle and the incident angle of ultrasonic sensors for the localization of a mobile robot have mainly focused on filtering or compensation methods. In this paper, the improved ultrasonic ranging calculation expression is presented, which is based on the original distance detection model for ultrasonic sensors. The ultrasonic sensor scanning method described herein can be used to recognize the inclined plate and acquire the position and posture of the ultrasonic sensor relative to the framework of the inclined plate. Finally, the omni-directional scanning localization method of a mobile robot is put forth based on this scanning method.

The organization of this paper is as follows: Section 2 analyses the affections of the divergence angle and the incidence angle on the ranging accuracy of the ultrasonic sensor, and deduces the improved algorithm expression. Section 3 introduces the methodology of the edge detection and the recognition of the inclined plate, and is extended in the application of the omni-directional scanning localization method in Section 4. In Section 5, the experiments of the identification of thresholds and the actual localization of a mobile robot are implemented, and the results verified the proposed localization methodology. Finally, Section 6 offers brief concluding comments.

## 2. The Divergence Angle and the Incidence Angle of an Ultrasonic Sensor 

**Figure 1:**
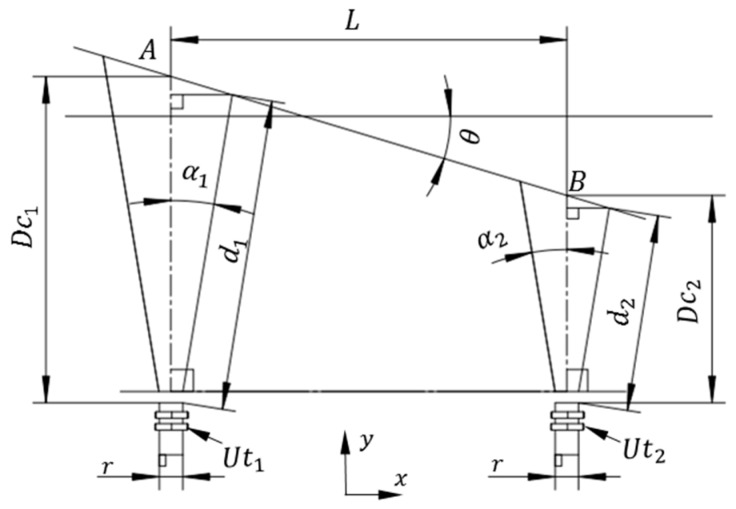
The divergence angle and the incidence angle.

In order to analyze the impact of the divergence angle and the incident angle on the measurement accuracy of an ultrasonic sensor, a geometric model is established as shown in Figure 1, where $Ut_{i}$ represents the ultrasonic sensor, i=(1,2) is the number of ultrasonic sensor, $\alpha_{i}$ represents the divergence angle of $Ut_{i}$ (in degree), AB is the reference plate, $d_{i}$ represents the direct distance measurement of $Ut_{i}$ (in mm), $Dc_{i}$ is the actual distance value (in mm) between $Ut_{i}$ and the reference plate *AB* in the *y-*direction, L is the distance (in mm) between two ultrasonic sensors in the *x*-direction and r is the diameter (in mm) of the ultrasonic sensor, $Ut_{i}$ . The incidence angle, θ , is defined as the angle between the cross-section of $Ut_{i}$ and the plane of the reference plate, *AB*, (in degree).

**Figure 2:**
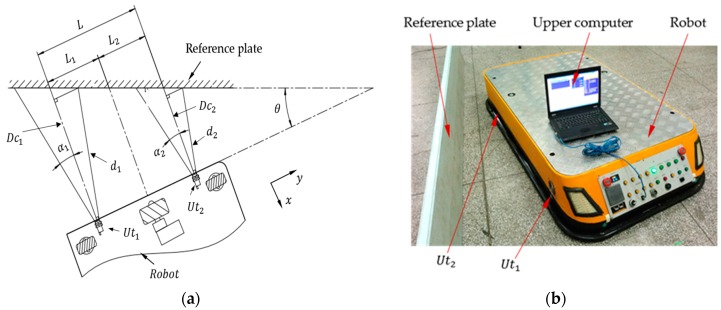
Lateral localization of a mobile robot. (**a**) The mathematical model of the localization application; (**b**) the practical application of lateral localization.

From Figure 1, it can be concluded that the actual distance value, $Dc_{i}$ , between the ultrasonic sensor, $Ut_{i}$ , and the reference plate, *AB*, in the *y*-direction can be expressed as:

(1) $$Dc_{i}=f\left(d_{i},\alpha_{i},\theta \right)=d_{i}C\left(\alpha_{i}\right)+\left[d_{i}S\left(\alpha_{i}\right)+\frac{r}{2}\right]T\left(\theta \right),$$

where $C\left(\alpha_{i}\right)$ is the cosine trigonometric function, $\cos\left(\alpha_{i}\right)$ , $S\left(\alpha_{i}\right)$ is the sine function, $\sin\left(\alpha_{i}\right)$ , T(θ) is the tangent function, tan(θ) , and i=(1,2) is the number of ultrasonic sensors. Conventionally, the direct distance, $d_{i}$ , is taken as the actual distance, $Dc_{i}$ . However, from the Equation (1), it is apparent that the actual distance, $Dc_{i}$ , is different from $d_{i}$ and is influenced by the divergence angle, $\alpha_{i}$ , and the incidence angle, θ , and that if these angles are ignored, the real distance measurement accuracy of the ultrasonic sensor would be affected. It is well known that the divergence angle, α , is an intrinsic property of a given ultrasonic sensor, which is invariant for a specified ultrasonic sensor but varies between different ultrasonic sensors, and can be obtained through experiments or from the factory manual. Usually, in most applications, the incidence angle, θ , is set to zero when a single ultrasonic sensor is used or in non-positioning and non-obstacle avoidance situations. However, when multiple ultrasonic sensors are used simultaneously and accurate localization is required, such as in the application presented in Figure 2, where two ultrasonic sensors are applied, the incidence angle, θ , can be expressed as shown in Equation (2):

(2) $$\theta =g\left(Dc_{1},Dc_{2}\right)=\tan^{-1}\left(\frac{Dc_{1}-Dc_{2}}{L}\right).$$

From Equations (1) and (2), the incidence angle, θ , can be described as:

(3) $$\theta =\{\begin{array}{c} \tan^{-1}\left[\frac{d_{1}C\left(\alpha_{1}\right)-d_{2}C\left(\alpha_{2}\right)}{L-d_{1}S\left(\alpha_{1}\right)+d_{2}S\left(\alpha_{2}\right)}\right]\;if(d_{1}>d_{2})\\ 0\;elseif\left(d_{1}=d_{2}\right)\\ \tan^{-1}\left[\frac{d_{2}C\left(\alpha_{2}\right)-d_{1}C\left(\alpha_{1}\right)}{L+d_{1}S\left(\alpha_{1}\right)-d_{2}S\left(\alpha_{2}\right)}\right]\;if(d_{1}<d_{2}) \end{array}.$$

## 3. Edge Detection and Recognition of the Inclined Plate

Earlier in this work, the effects of the divergence angle and the incidence angle on the accuracy of the distance measurement of an ultrasonic sensor have been analyzed and a novel ranging algorithm with consideration of the divergence angle and the incidence angle of the ultrasonic sensor has been established. Now, this conclusion is going to be executed to the edge detection of an inclined plate. In order to enable object identification in a given environment, Zhong et al. [21] determined the range, bearing angle and shape (edge or plane) of objects from a single measurement of a robot using a single transmitter and a multi-receiver of ultrasonic sensors. Ohtani and Baba [22] designed a prototype system for the shape recognition, position and posture measurement of an object, using an ultrasonic sensor array made up of multi ultrasonic transmitters and receivers arranged in the same plane, a processing unit and a neural network. Although both of these studies could recognize objects and detect the edge of an object, the transmitter and the receiver of each ultrasonic sensor are separate, with the transmitter irradiating the measured object with ultrasonic waves and the receiver picking up the reflected waves to recognize the object. This method requires multiple positions for the transmitters and the receivers, which needs more space and is inflexible. Therefore, it is quite significant to integrate the transmitter and the receiver of an ultrasonic sensor by controlling the scanning of the ultrasonic sensor to achieve the identification of objects.

The ultrasonic sensors used in this paper are all the integrated ones, and they all have the time synchronization (avoiding the mutual interference between different ultrasonic sensors) and temperature compensation functions. The minimal detection distance of an ultrasonic sensor (dead zone) is denoted as $D_{\min}$ , and the maximal as $D_{\max}$ , the actual distance between the object and the ultrasonic sensor as *D*, where $D\in \left[D_{\min\;},D_{\max}\right]$ to ensure that the distance value, *D*, is applicable and reliable. When the direction of the ultrasonic sensor relative to the inclined plate scans continuously, relative changes in the value measured by the ultrasonic sensor occur, whereas, at the edge of the inclined plate, the measured value oscillates irregularly. Thus, we define a threshold ξ to determine whether the edge of the inclined plate is detected or not, where ξ is a small positive real number. Let $d_{i}$ and $d_{i+1}$ be the two successive measurement record values of a specific edge, with i=1, 2, …, n , where n is the total number of record groups. If $\Delta d=\|d_{i}-d_{i+1}\|$ satisfies Equation (4), it is definitely accounted for that the edge of the inclined plate is detected and the distance from the ultrasonic sensor to the edge of inclined plate is $d_{i}$ :

(4) $$\Delta d=\|d_{i}-d_{i+1}\|<\xi .$$

**Figure 3:**
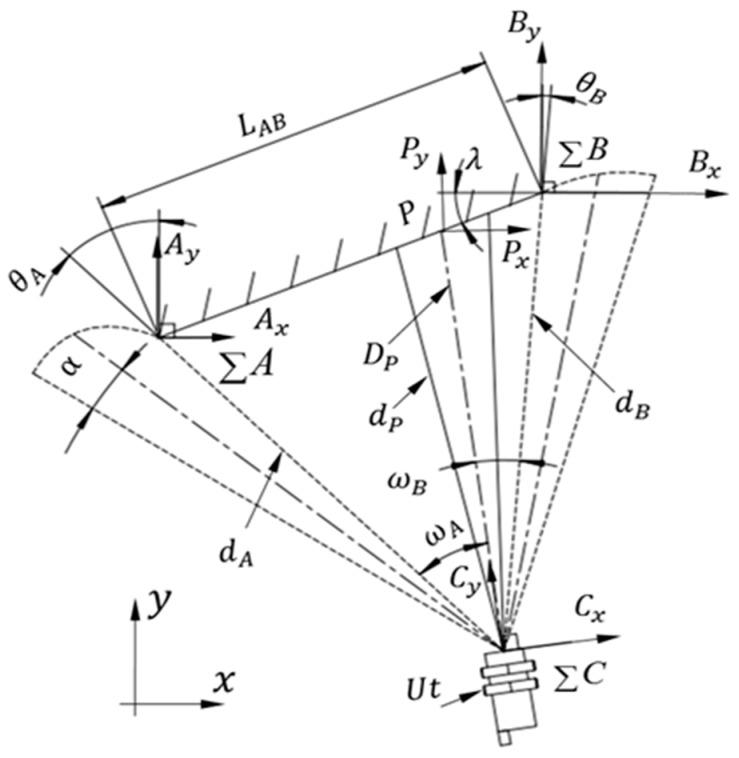
Edge detection of the inclined plate.

The edge detection model is as shown in Figure 3, where α is the divergence angle of the ultrasonic sensor Ut , ∑C is the coordinate system of Ut , $L_{AB}$ is the actual length of the inclined plate AB , and ∑A and ∑B are the coordinate systems at point A and B , respectively. λ is the angle between the inclined plate AB and the horizontal direction of the *x*-axis, $D_{P}=f\left(d_{P},\alpha ,\theta \right)$ is the actual distance from the ultrasonic sensor, Ut , to the point, P , on the inclined plate. Correspondingly, $d_{P}$ is the direct distance measurement of point P , $d_{A}$ and $d_{B}$ are the directly measured values of points *A* and *B*, respectively, while scanning the plate *AB*, and can all be confirmed from Equation (4). $\omega_{1}$ and $\omega_{2}$ are the rotation angles of the ultrasonic sensor scanning from point *P* to point *A* counterclockwise and scanning from point *P* to point *B* clockwise, respectively, where $\omega =\omega_{A}+\omega_{B}$ . The theoretical length of plate *AB*, $l_{AB}$ , is obtained from $d_{A}$ , $d_{B}$ and ω , which are contained in the triangle ∆ABC:

(5) $$l_{AB}=\sqrt{{\left(d_{A}\right)}^{2}+\left(d_{B}\right)-2d_{A}d_{B}C\left(\omega \right)},$$

(6) $$\Delta L=\left|l_{AB}-L_{AB}\right|<\delta .$$

In Equation (6), δ is a positive number, given as the length recognition threshold of the inclined plate. The values, $d_{A}$ , $d_{B}$ , $\omega_{A}$ and $\omega_{B}$ , acquired from Equation (4), are considered to be correct if $l_{AB}$ satisfies Equation (6). Otherwise, scanning is repeated until Equation (6) is satisfied and the plate is recognized. Set $\theta_{A}$ as the angle between the straight line *AC* and the *y*-direction of the reference frame ∑A , $\theta_{P}$ as the angle between the straight line *PC* and the *y*-direction of the reference frame ∑P , $\theta_{B}$ as the angle between the straight line *BC* and the *y*-direction of the reference frame ∑B . According to the homogeneous coordinate transformation methodology, the position and posture, TPA and TPB , of system ∑P relative to ∑A and ∑B , respectively, are obtained as:

(7) TPA=[EdAS(θA)−dAC(θA)001]=[Rz(θA−ωA−α−θP)dAS(θA)−dAC(θA)001],

(8) TPB=[E−dBS(θB)−dBC(θB)001]=[Rz(ωB−θB+α−θP)−dBS(θB)−dBC(θB)001],

where E is a 3×3 unit matrix, and $R_{z}\left(\theta \right)$ is a rotation matrix as shown in Equation (10). The angles, $\theta_{A}$ and $\theta_{B}$ , in Equations (7) and (8) are as shown in Equation (9):

(9) $$\{\begin{array}{c} \theta_{A}=\frac{\pi }{2}-sin^{-1}\left(\frac{d_{B}}{L_{AB}}S\left(\omega \right)\right)+\lambda \\ \theta_{B}=\frac{\pi }{2}-sin^{-1}\left(\frac{d_{A}}{L_{AB}}S\left(\omega \right)\right)-\lambda \end{array},$$

(10) $$R_{z}\left(\theta \right)=\left[\begin{array}{ccc} C\left(\theta \right) & -S\left(\theta \right) & 0\\ S\left(\theta \right) & C\left(\theta \right) & 0\\ 0 & 0 & 1 \end{array}\right].$$

According to the earlier definition, the incidence angles between the ultrasonic sensor, Ut , and the inclined plate, *AB*, at point *P* can be described as θPA and θPB , respectively, relative to the reference frames ∑A and ∑B :

(11) {θPA=ωA+α−θA+λθPB=θB−α−ωB+λ.

Through a review of the above studies, the position (as shown in Equation (7) or (8)) and posture (as shown in Equation (11)) of an ultrasonic sensor relative to a fixed plate can be obtained. Similarly, if the ultrasonic sensor is installed on a mobile robot, the scanning of the ultrasonic sensor is accomplished by the rotation of the robot. Thus, the position and posture of the mobile robot can also be acquired.

## 4. Omni-Directional Scanning Localization Method

**Figure 4:**
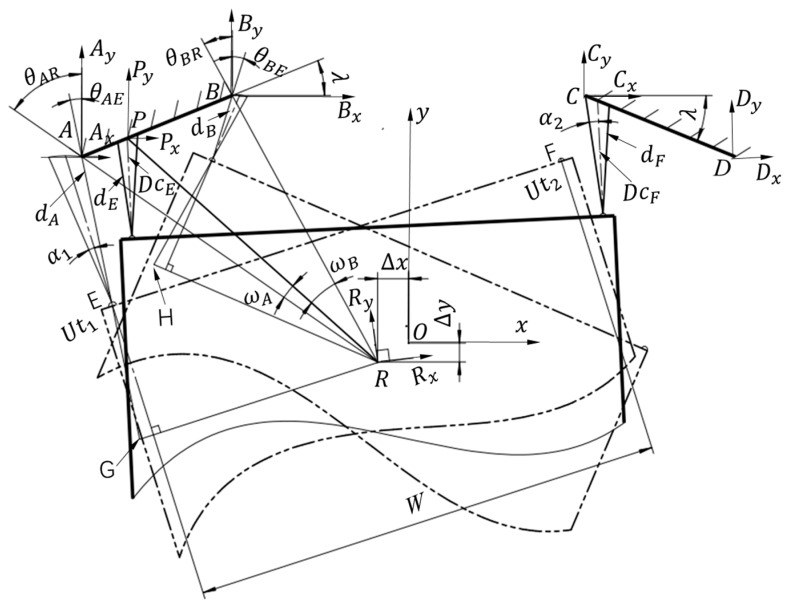
Localization model of the mobile robot.

The above section introduced the ultrasonic sensor scanning recognition method of a fixed inclined plate and deduced the position and posture of the ultrasonic sensor relative to the plate. However, in most practical applications, the ultrasonic sensor is installed on a mobile robot, the rotation center of the sensor is the center of the robot and the ultrasonic sensor scans the objects along with the spin of the robot. To further research the localization method of a mobile robot with an ultrasonic sensor, a model is established as shown in Figure 4.

In Figure 4, a coordinate system parallel to the reference frame ∑O at points *A*, *B*, *C*, *D* and *P* as ∑A , ∑B , ∑C , ∑D and ∑P is established, as is the robot base reference frame, ∑R , at the center of mobile robot, whose axes are parallel to the outlines of the robot. *AB* and *CD* are the two inclined plates fixed on the localization worksite or the pallet to be carried away by the robot, the angle between plate *AB* and the horizontal direction of the *x*-axis is λ , and the angle between plate *CD* and the horizontal direction of the *x*-axis is −λ . *R* is the center of the mobile robot body. The two ultrasonic sensors, $Ut_{1}$ and $Ut_{2}$ , are installed at the two points, *E* and *F*, on the front of the robot body, characterized by the divergence angles, $\alpha_{1}$ and $\alpha_{2}$ , respectively. The distance parallel to the transverse of the robot between $Ut_{1}$ and $Ut_{2}$ is *W*. $d_{E}$ is the directly measured value of $Ut_{1}$ when the robot is located at position *R*, and $Dc_{E}$ is the actual one. Similarly, $d_{F}$ is the directional measurement of $Ut_{2}$ and $Dc_{F}$ is the actual one. *P* is the reflection point at the present position and posture of $Ut_{1}$ . 

The desired distance between the ultrasonic sensors, $Ut_{1}$ and $Ut_{2}$ , and the plate, *AB* and *CD*, is denoted as *D* and the posture of the robot is $\theta_{O}=0^{{}^{\circ}}$ when the robot is at the reference position *O*. The inclined plate *AB* and *CD* are symmetrically arranged to the *y*-axis of the frame ∑O . The position and posture error of the robot at the point *R* relative to the reference frame, ∑O , is given as $\Delta \varepsilon ={\left[{\Delta \varepsilon }_{x},{\Delta \varepsilon }_{y},{\Delta \varepsilon }_{\theta }\right]}^{T}.$ The position of point *E* in the frame ∑R , PER , is PER=[Ex,Ey]T . The distance from point *A* to point *D* is $L_{AD}$ at the horizontal direction of the *x*-axis, and the distance from point *B* to point *C* is $L_{BC}$ at the horizontal direction of the *x*-axis.

First, the robot is controlled to point *O* manually and it is made sure that $Dc_{E}=Dc_{F}=D$ . Then, the robot rotation is controlled around its center, and, using the scanning recognition method introduced in Section 3, the counterclockwise and clockwise angles of the robot from point *P* to point *A* and point *B*, $\omega_{AO}$ and $\omega_{BO}$ , respectively, are recorded. Finally, the homogeneous coordinate transformations, ToA and ToB , from the coordinate system ∑A and ∑B to the coordinate system ∑P is accomplished as follows:

(12) TOA=[Rz(θAO−ωAO−α1)POA01]=[E−LAD2Ey+D−LAD−W2T(λ)001],

(13) TOB=[Rz(θBO+ωBO+α1)POB01]=[E−LBC2Ey+D+LBC−W2T(λ)001],

where $\theta_{AO}$ is the angle between the straight line *AO* and the *y*-axis of the reference frame ∑A , and $\theta_{BO}$ is the angle between the straight line *BO* and the *y*-axis of the reference frame ∑B . From Equations (12) and (13), the distance from point *O* to point *A* and point *B*, $L_{AO}$ and $L_{BO}$ , are as follows:

(14) LAO=(POxA)2+(POyA)2,

(15) LBO=(POxB)2+(POyB)2,

(16) $$\omega_{O}=\omega_{AO}+\omega_{BO}.$$

The theoretical length $l_{AB}$ of the plate AB in the triangle ∆*ABO* is calculated from the values of $L_{AO}$ , $L_{BO}$ and $\omega_{O}$ :

(17) $$l_{AB}=\sqrt{{\left(L_{AO}\right)}^{2}+{\left(L_{BO}\right)}^{2}-2L_{AO}L_{BO}C\left(\omega_{O}\right)}.$$

In accordance with the definition of the incidence angle, the incidence angle, θ , relative to the reference frames ∑A and ∑B is:

(18) θ=θPA=θPB=λ .

Similarly, the transformation of the robot at the position *R* relative to ∑A and ∑B are TRA and TRB , respectively:

(19) TRA=[Rz(θAR−ωA−α1)LARS(θAR)−LARC(θAR)001],

(20) TRB=[Rz(θBR+ωB+α1)LBRS(θBR)−LBRC(θBR)001],

where $\theta_{AR}$ is the angle between the straight line *AR* and the *y*-axis of the reference frame ∑A , and $\theta_{BR}$ is the angle between the straight line *BR* and the *y*-axis of the reference frame ∑B :

(21) $$\{\begin{array}{c} \theta_{AR}=\frac{\pi }{2}-\sin^{-1}\left(\frac{L_{BR}}{L_{AB}}S\left(\omega \right)\right)+\lambda \;\\ \theta_{BR}=-\frac{\pi }{2}+\sin^{-1}\left(\frac{L_{AR}}{L_{AB}}S\left(\omega \right)\right)+\lambda \end{array},$$

(22) $$\{\begin{array}{c} L_{AR}=\sqrt{{\left(L_{AG}\right)}^{2}+{\left(L_{GR}\right)}^{2}-2L_{AG}L_{GR}S\left(\alpha_{1}\right)}\\ L_{BR}=\sqrt{{\left(L_{AH}\right)}^{2}+{\left(L_{HR}\right)}^{2}-2L_{AH}L_{HR}S\left(\alpha_{1}\right)} \end{array},$$

(23) {LAG=dA+PRyEC(α1)LGR=PRyET(α1)+PRxELAH=dB+PRyEC(α1)LHR=LAH.

The theoretical length of the plate *AB*, $l_{AB}$ , can be deduced in the triangle ∆*ABR* as:

(24) $$l_{AB}=\sqrt{{\left(L_{AR}\right)}^{2}+\left(L_{BR}\right)-2L_{AR}L_{BR}C\left(\omega \right)}.$$

The divergence angles, θPA and θPB , of the ultrasonic sensor are listed in the Equation (25):

(25) {θPA=ωA+α1−θAR+λθPB=λ−θBR−ωB−α1.

The position and posture error of the robot at the position *R* relative to the position O in reference to ∑A and ∑B are ΔOAεR and ΔOBεR :

(26) ΔOAεR=[ΔOAεxRΔOAεyRΔOAεθR]=[PRxA−POxAPRyA−POyARRxA−ROxA],

(27) ΔOBεR=[ΔOBεxRΔOBεyRΔOBεθR]=[PRxB−POxBPRyB−POyBRRxB−ROxB].

Considering Equations (12)–(15), (19), (20), (26) and (27), the errors ΔAεR and ΔAεR are calculated as follows:

(28) ΔOAεR=12[2LARS(θAR)+LAD(LAD−W)T(λ)−2(Ey+D+LARC(θAR))2(θAR−ωA−α1)],

(29) ΔOBεR=12[2LBRS(θBR)+LBC−(LBC−W)T(λ)−2(Ey+D+LBRC(θBR))2(θBR+ωB+α1)].

The position and posture error thresholds of the robot at position *R* are set to $\varepsilon ={\left[\varepsilon_{x},\varepsilon_{y},\varepsilon_{\theta }\right]}^{T}$ . The robot meets the localization requirement if the error Δε satisfies the Equation (30):

(30) $$\Delta \varepsilon =\left\|[\begin{array}{c} \Delta \varepsilon_{x}\\ \Delta \varepsilon_{y}\\ \Delta \varepsilon_{\theta } \end{array}]\right\|<\left[\begin{array}{c} \varepsilon_{x}\\ \varepsilon_{y}\\ \varepsilon_{\theta } \end{array}\right]=\varepsilon .$$

Up until this stage, the position and posture and the error of the mobile robot relative to the reference frame, ∑A and ∑B , at the points *A* and *B* have been acquired. For the actual application, the localization of the robot is achieved according to the follow steps.

**Step** **1** Preparation. First, the positions of the inclined plate *AB* and *CD* are set up, and their lengths and poses are specified. Then, values of thresholds ξ , δ and ε are assigned. Next, the distance measured value *D* of ultrasonic sensor at the reference pose *O* is verified, and the transform position and posture TOA and TOB are calculated.
**Step** **2** Satisfaction of pre-localization condition. The robot moves from somewhere to the control point *R*, which can be anywhere, and whether the actual distance measurement values $Dc_{E}$ and $Dc_{F}$ satisfy $\|Dc_{E}-D\|<\rho_{E}$ and $\|Dc_{F}-D\|<\rho_{F}$ , with $\rho_{E}$ and $\rho_{F}$ being positive and real numbers, are judged. If these prerequisites are met, the directly measured distances $d_{A}$ and $d_{B}$ of the ultrasonic sensor $Ut_{1}$ , and the rotation angles $\omega_{A}$ and $\omega_{B}$ of the robot are recorded and it is possible to proceed to Step 3. Otherwise, the process must be repeated until the pre-localization conditions are met.
**Step** **3** Edge detection of inclined plate. Along with the spin of the robot around its center, the ultrasonic sensor $Ut_{1}$ scans the inclined plate *AB*, and the distance $d_{A}$ and $d_{B}$ of the edge of plate *AB* is measured. If $d_{A}$ and $d_{B}$ satisfy the Equation (4), it is possible go to the subsequent step. If not, re-scanning is necessary.
**Step** **4** Verification of the length of the inclined plate. The theoretical length $l_{AB}$ of plate *AB* is calculated through Equation (24). If Equation (6) is satisfied, proceed to Step 5. If not, it is necessary to return to Step 2 and repeat Steps 2–4.
**Step** **5** Calculation of the position and posture of the robot. The position and posture, TRA and TRB , of the mobile robot relative to the reference frame ∑A and ∑B are calculated, respectively, through Equations (19) and (20).
**Step** **6** Satisfaction of localization requirement. The position and posture errors, ΔAεR and ΔBεR , of the robot are computed. If ΔAεR and ΔBεR satisfy Equation (30), the localization requirement is achieved. Otherwise, return to Step 2 and repeat Steps 2–6.

## 5. Threshold and Experiments

### 5.1. Threshold Identification Experiment

The omni-directional scanning localization method of a mobile robot and its application steps have been introduced. However, the confirmation of the different threshold values defined in the method may affect the accuracy of the localization.

The most important one is the plate edge detection threshold, ξ , which decides the sink or swim of the edge detection of the plate, and affects the correctness of the incidence angle of the ultrasonic sensor and the theoretical length of the plate. Therefore, it is necessary to get the precise value of ξ .

**Figure 5:**
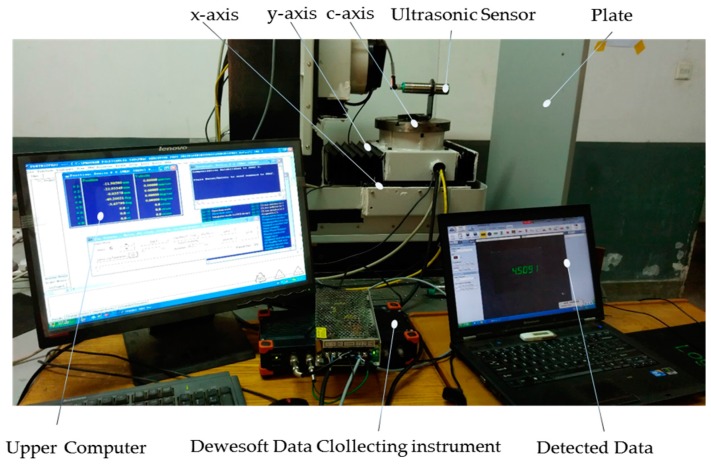
Threshold of edge detection experiment.

**Figure 6:**
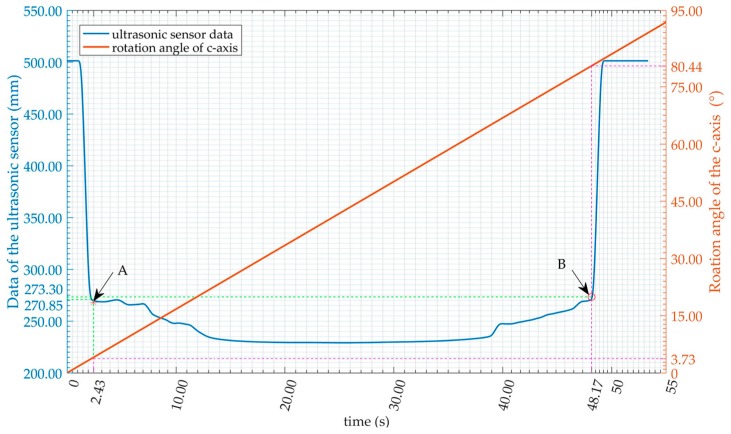
Data of edge detection.

The experiment preformed to confirm the edge detection threshold, ξ , is as shown in Figure 5, where the ultrasonic sensor is mounted on the *c*-axis of the independently researched and developed precision five-axis machine, and the *x*-axis of the machine is assembled at the top of the *y*-axis and below the *c*-axis. The scanning movement of the ultrasonic sensor is driven by the CNC (Computer Numerical Control) programing of the *x*-axis, the *y*-axis and the rotation of the *c*-axis of the machine. The plate edge data measured by the ultrasonic sensor is recorded by the DAQ (Date Acquisation), SIRISI-8A (DEWESoft, Kumberg, Austria) as shown in Figure 6.

In Figure 6, the blue line is the recorded data of the ultrasonic sensor, the red line is the rotation angle of the *c*-axis of the machine, points *A* and *B* are the detected edge of the inclined plate, their vertical data, $d_{A}$ and $d_{B}$ , are the detected distances of the two sides of the plate, and ω is the rotation angle of the *c*-axis from point *A* to point *B*. The theoretical length of plate $l_{AB}$ can be calculated using Equation (5) and the length error ΔL from Equation (6). After many experiments and much analysis, it has been found that the error ΔL increases sharply when ξ<0.008 and at a faster rate when ξ>0.010 . Therefore, ξ=0.009 is concluded as the edge detection threshold and δ=3 as the length recognition threshold. The ranging threshold $\rho_{E}=100$ and $\rho_{F}$ =100. The position and posture error threshold ε can be determined empirically as $\varepsilon ={\left[10\;\mathrm{mm},5\;\mathrm{mm},1^{{}^{\circ}}\right]}^{T}$ .

### 5.2. Localization Experiment

**Figure 7:**
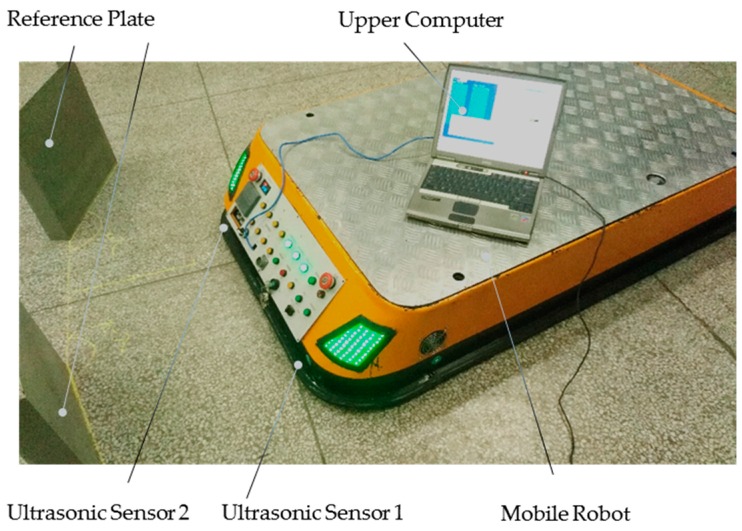
The localization experiment.

The localization experiment has been implemented on the independently developed latent and towing mobile robot as shown in Figure 7. The mobile robot is a differential driving robot, with two driving wheels mounted coaxially on the left and right sides of the robot symmetrically and four universal wheels distributed at the four corners of the robot correspondingly. In order to ensure that the six wheels of the robot can be in better contact with the ground while driving at the same time, the universal wheels have been designed with elastic suspension structure. The maximum speed of the robot is 0.5 m/s, the maximum loading capacity is 2 tons, and its length, width and height are 1560 mm, 900 mm, and 300 mm, respectively.

Parts of the experimental localization data are listed in Table 1. $Dc_{1}$ and $Dc_{2}$ are the actual distances measured from the ultrasonic sensors $Ut_{1}$ and $Ut_{2}$ , respectively. $\Delta \varepsilon_{x}$ , $\Delta \varepsilon_{y}$ and $\Delta \varepsilon_{\theta }$ are the localization error of the robot relative to the *x*, *y* and *c* axes, of the reference frame ∑O .

As can be seen from Table 1, the localization accuracy of the robot is $\Delta \varepsilon_{y}\leq \pm 3.33$ mm on the *y*-axis (the front of the robot), $\Delta \varepsilon_{x}\leq \pm 6.21$ mm on the *x*-axis (the lateral of the robot), $\Delta \varepsilon_{\theta }\leq \pm 0.20^{{}^{\circ}}$ on the *c*-axis (the posture of the robot). The localization accuracy, Δε , satisfies the localization error threshold, ε , and these results verify the efficacy of the omni-directional scanning localization method.

To this step, the methodology of the edge detection and recognition of an inclined plate and the omni-directional scanning localization of a robot is verified by the above experiments, and the thresholds of the omni-directional scanning localization method are confirmed through experiments and experience. At the end, the novel ranging algorithm of an ultrasonic sensor is certified. The localization accuracy of the proposed omni-directional scanning method is suitable for a variety of applications, and several application examples are described in the next section.

### 5.3. Discussion and Application

**Figure 8:**
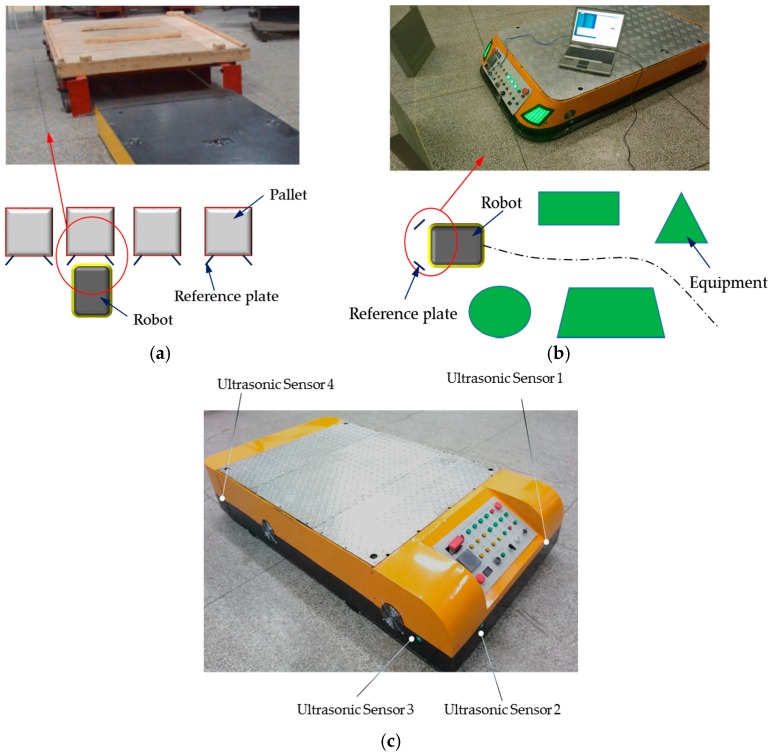
Applications of the omni-directional scanning localization method. (**a**) The transit tasks application; (**b**) the localization application; (**c**) the application of an omni-directional mobile.

The proposed localization method, an omni-directional localization method which can realize the forward, lateral and posture localization of a robot simultaneously, is different from the localization application as shown in Figure 2, which can only achieve the lateral (or the front) and posture position of the robot. This method takes up little space and is more convenient in the localization applications such as those shown in Figure 8, where Figure 8a is a transit task application of a busy factory (this is a case of a textile enterprise), in which there are lots of shelves arranged, according to certain rules, and they are waiting to be carried away. Figure 8b is another localization application (this is a case of a rice winery), where the robot continuously moves from anywhere to the tight localization site in a continuous path and confirms its position and posture. 

The proposed localization method can also be leveraged for other types of robots, if the robot satisfies the localization conditions, such as the omni-directional mobile robot shown in Figure 8c, for which each of the four sides of the robot installed two ultrasonic sensors to ensure the realization of the localization on any one side of the robot. The robot has four Mecanum’s wheels, with which the robot can move smoothly in *x*, *y* and *c* directions [23]. Further research and application of this technology is still ongoing, such as the multi-robot localization in the dynamic environment. 

## 6. Conclusions

First, the novel ranging algorithm of an ultrasonic sensor is established by simultaneously considering both the divergence angle and the incidence angle, which improved the accuracy of the measurement of the ultrasonic sensor.

Second, the edge detection and recognition of an inclined plate is introduced by using the proposed ranging algorithm, based on which the position and posture of an ultrasonic sensor relative to the plate are obtained. 

Third, the ultrasonic sensor is installed on a mobile robot, and the positioning method of the ultrasonic sensor is extended to the omni-directional scanning localization of a mobile robot to achieve the forward, lateral and posture localizations synchronously. Details of the localization methodology are introduced and discussed, and the application steps are summarized.

Finally, the thresholds of the localization method are confirmed through experiments and experience, and the omni-directional scanning localization method is verified by the localization experiment of a differential driving robot. The application of the method on other types of mobile robots is discussed and several real applications are given. 

Finally, the main concern of the proposed localization method is the local localization of a mobile robot at the worksite. Further research and application of this technology is still ongoing, such as the combination of the local and the global localization of a robot, and the research of applications of the proposed localization method for multi-robots with multi-sensor information fusion technology in dynamic circumstances.

## Figures and Tables

**Table 1:** The localization data of the robot.

$D_{c1}$ (mm)	$D_{c2}$ (mm)	$\Delta \varepsilon_{x}$ (mm)	$\Delta \varepsilon_{y}$ (mm)	$\Delta \varepsilon_{\theta }$ (°)
298.78	300.42	−4.65	−0.40	−0.16
300.19	300.57	−1.08	0.38	−0.04
301.14	301.18	−0.11	1.16	−0.00
300.28	300.64	−1.02	0.46	−0.03
302.31	304.35	−5.78	3.33	−0.19
298.79	300.98	−6.21	−0.12	−0.20
300.84	299.50	3.80	0.17	0.13
297.98	297.41	1.62	−2.31	0.05
